# Key Aspects of Neurovascular Control Mediated by Specific Populations of Inhibitory Cortical Interneurons

**DOI:** 10.1093/cercor/bhz251

**Published:** 2019-11-20

**Authors:** L Lee, L Boorman, E Glendenning, C Christmas, P Sharp, P Redgrave, O Shabir, E Bracci, J Berwick, C Howarth

**Affiliations:** Department of Psychology, University of Sheffield, Sheffield S1 2LT, UK

**Keywords:** BOLD fMRI, neurovascular coupling, nitric oxide synthase, optogenetics, somatostatin

## Abstract

Inhibitory interneurons can evoke vasodilation and vasoconstriction, making them potential cellular drivers of neurovascular coupling. However, the specific regulatory roles played by particular interneuron subpopulations remain unclear. Our purpose was therefore to adopt a cell-specific optogenetic approach to investigate how somatostatin (SST) and neuronal nitric oxide synthase (nNOS)-expressing interneurons might influence the neurovascular relationship. In mice, specific activation of SST- or nNOS-interneurons was sufficient to evoke hemodynamic changes. In the case of nNOS-interneurons, robust hemodynamic changes occurred with minimal changes in neural activity, suggesting that the ability of blood oxygen level dependent functional magnetic resonance imaging (BOLD fMRI) to reliably reflect changes in neuronal activity may be dependent on type of neuron recruited. Conversely, activation of SST-interneurons produced robust changes in evoked neural activity with shallow cortical excitation and pronounced deep layer cortical inhibition. Prolonged activation of SST-interneurons often resulted in an increase in blood volume in the centrally activated area with an accompanying decrease in blood volume in the surrounding brain regions, analogous to the negative BOLD signal. These results demonstrate the role of specific populations of cortical interneurons in the active control of neurovascular function.

## Introduction

Neurovascular coupling (NVC) is the mechanism through which local cerebral blood flow (CBF) changes are tightly coupled to increases in neural activity (Roy and Sherrington 1890). Since the reserves of oxygen and glucose within neurons are strictly limited, such coupling is essential for normal brain function (Buxton 2010; Leithner and Royl 2014). Variations in blood oxygenation and volume evoked by neural activity underlie functional imaging signals, such as blood oxygen level dependent functional magnetic resonance imaging (BOLD-fMRI, [Ogawa et al. 1990]), which are commonly used as a surrogate measure of local changes in neuronal activity. While most research has focused on the ability of excitatory neurons (Lecrux et al. 2011; Lacroix et al. 2015) and astrocytes (Zonta et al. 2003; Mulligan and MacVicar 2004; Lind et al. 2013) to elicit changes in CBF, there has been less focus on the role of inhibitory neurons. GABAergic interneurons innervate local microvessels (Vaucher et al. 2000; Cauli et al. 2004; Hamel 2006) and have been shown to induce both vasodilation and constriction (Cauli et al. 2004) making them potential cellular drivers of NVC.

Although recent studies have investigated the contribution of inhibitory interneurons to CBF regulation by using an optogenetic approach targeting VGAT (vesicular GABA transporter)-expressing neurons (Anenberg et al. 2015; Uhlirova et al. 2016; Vazquez et al. 2018), the role of specific subpopulations of GABAergic interneurons remains unknown. Somatostatin (SST)-expressing neurons, which account for around 30% of GABAergic interneurons in the somatosensory cortex (Rudy et al. 2011), contact brain microvessels, in particular those in the superficial layers of the cortex (Kocharyan et al. 2008). The release of GABA by SST interneurons has been suggested to contribute to basal forebrain stimulation-evoked cortical CBF responses (Kocharyan et al. 2008). In addition, approximately 28% of GABAergic neurons (Cauli et al. 2004), including a small subset of SST interneurons predominantly located in cortical layers II/III and V/VI (Karagiannis et al. 2009; Yavorska and Wehr 2016), express neuronal nitric oxide synthase (nNOS). This further subpopulation of interneurons releases nitric oxide (NO), which for a long time has been known to be a potent vasodilator (Furchgott and Zawadzki 1980; Ignarro et al. 1987; Palmer et al. 1987). nNOS interneurons are therefore of particular interest in terms of CBF regulation. To investigate the role of these two subpopulations of GABAergic interneuron, we used a cell-type-specific optogenetic approach that specifically targeted SST- or nNOS-expressing interneurons. By separately activating these two subsets of inhibitory interneurons (those expressing SST or nNOS), we sought to determine how they might regulate cortical hemodynamics. We were able to show that activating both subsets of interneurons evoked a localized hemodynamic response. Importantly, in the case of optogenetic-activation of nNOS interneurons, the observed hemodynamic changes occurred with only a minimal change in measured multiunit neural activity. Alternatively, after activating SST interneurons negative hemodynamic responses were observed in the cortical areas surrounding the local area of optogenetic stimulation. This observation is similar to reported negative BOLD fMRI responses, which have been linked to inhibitory neuron activity (Shmuel et al. 2002, 2006; Stefanovic et al. 2004; Boorman et al. 2010, 2015). These observations suggest that specific subpopulations of cortical GABAergic interneurons have specific roles in NVC. Also, that the ability of BOLD signals to act as a surrogate measure of local neural activation may in part be dependent upon which subpopulation of neurons are being activated.

## Materials and Methods

### Animals

All animal procedures were performed in accordance with the guidelines and regulations of the UK Government, Animals (Scientific Procedures) Act 1986 and approved by the University of Sheffield Ethical review and licensing committee. Mice had ad libitum access to food and water and were housed on a 12 h dark/light cycle. We used 39 mice of both sexes including 18 nNOS-CreER × ChR2-EYFP mice (M/F, 19–33 g), 12 Sstm2.1Crezjh/j × ChR2-EYFP mice (M/F, 22–44 g), 5 C57Bl/6J mice (F, 23–25 g), and 4 non-ChR2-expressing littermates of nNOS-ChR2 mice (M, 33–39 g). Sstm2.1Crezjh/j × ChR2-EYFP (SST-ChR2) mice were obtained by crossing homozygous SOM-IRES-Cre mice (Stock 013044, Jackson Laboratory [Network NNBCD 2009; Taniguchi et al. 2011]) with homozygous ChR2(H134R)-EYFP mice (Stock 024109, Jackson Laboratory [Madisen et al. 2012]), as described previously (Elghaba et al. 2016).

The nNOS-CreER × ChR2-EYFP (nNOS-ChR2) mice were obtained by crossing heterozygous nNOS-CreER (Stock 014541, Jackson Laboratory [Taniguchi et al. 2011]) with homozygous Ai32 mice (Stock 024109, Jackson Laboratory [Madisen et al. 2012]). Littermates lacking the nNOS-CreER insertion do not express ChR2 and were used as control mice in this study. ChR2 expression was induced by intraperitoneal (IP) injection of tamoxifen (Sigma-Aldrich) at 100 mg/kg, administered three times over 5 days. Treatment with tamoxifen was carried out when mice were aged between 1 and 2 months old, this took place a minimum of 2 weeks prior to surgery to allow for gene expression to take place.

### Preparation of Chronic Cranial Window

A thinned cranial window was prepared over the right whisker barrel cortex, as described by Sharp et al. (2015). Anesthesia was induced through IP injection of fentanyl-fluanisone (Hypnorm, Vetapharm Ltd), midazolam (Hypnovel, Roche Ltd), and sterile water (in a ratio of 1:1:2 by volume; 7 mL/kg) and maintained using isoflurane (0.5–0.8%) in 100% oxygen at a flow rate of 1 L/min. All surgeries were carried out in a dark room using a surgical illuminator with a band pass filter (577 ± 5 nm) to avoid erroneous optogenetic activation in the ChR2-expressing mice. Mice were placed on a stereotaxic frame (Kopf Instruments), on a homeothermic blanket (Harvard Apparatus) maintaining rectal temperature at 37 °C. The bone overlying the right somatosensory cortex was thinned to translucency using a dental drill, forming a ≈3 mm^2^ optical window. A thin layer of clear cyanoacrylate was applied and a stainless steel head plate secured to the skull using dental cement (Super bond C&B, Sun Medical). Surgery was performed at least 2 weeks before the first experimental imaging session.

For experiments, anesthesia was induced as described above and maintained using isoflurane (0.25–0.7%) in 100% oxygen at a flow rate of 0.8 L/min. Mice were placed on a stereotaxic frame and head fixed via their head plate. Animals were placed on a homeothermic blanket maintaining rectal temperature at 37 °C.

### 2-Dimensional optical imaging spectroscopy

Animals underwent two experimental sessions. Session one, at least 2 weeks post-surgery, involved 2-D optical imaging spectroscopy (2D-OIS) recordings and application of both short and long duration whisker and light stimulations. Session 2, occurring at least 1 week after session 1, involved concurrent electrophysiology and 2D-OIS recordings, while applying both short and long duration stimulations (see Stimulations, below, for full details).

As described previously (Berwick et al. 2005), 2D-OIS was used to record changes in cortical hemodynamics, allowing the estimation of changes in cortical oxyhemoglobin (HbO_2_), deoxyhemoglobin (Hbr), and total hemoglobin concentration (Hbt). The cortex was illuminated with four wavelengths of light (587 ± 9, 595 ± 5, 560 ± 15, and 575 ± 5 nm) using a Lambda DG-4 high-speed filter changer (Sutter Instrument Company). The re-emitted light was collected at a frame rate of 32 Hz using a Dalso 1M60 CCD camera which was synchronized to the filter switching, thus producing an effective frame rate of 8 Hz. The camera was fitted with a 490 nm high pass filter to prevent light from the photostimulation LED being collected with the re-emitted light. The spatial maps recorded from the re-emitted light then underwent spectral analysis based upon the path length scaling algorithm described previously (Mayhew et al. 1999; Berwick et al. 2005). In brief, the algorithm uses a modified Beer–Lambert Law with a path length correction factor to convert detected attenuation in the re-emitted light into predicted absorption. These absorption values were then used to generate estimates of the changes in HbO_2_, Hbr, and Hbt from baseline values. The concentration of hemoglobin in tissue was assumed to be 100 μM and oxygen saturation was assumed to be 70%. This spectral analysis produced 2D images of the micromolar changes in volume of HbO_2_, Hbr, and Hbt over time.

### Stimulations

Whisker stimulation was performed using a plastic T-bar attached to a stepper motor, which deflected the whiskers ≈1 cm in the rostro-caudal direction (Sharp et al. 2015). Whiskers were deflected at 5 Hz for either 2 or 16 s. To improve the signal-to-noise for each experiment, whisker stimulation was presented 30 times in trials lasting 15–25 s each (2s stimulation) or presented 15 times in trials lasting 70 s each (16s stimulation).

Photostimulation was performed using a fiber-coupled LED light source (470 nm, ThorLabs) and was delivered to the point of activation using a fiber optic (core diameter 200 μm, ThorLabs). The cortex was illuminated for 2 s (pulse width 10 ms, 20 Hz, 2 V, 0.78 mW or 99 Hz, 1 V, 0.45 mW) or 16s (pulse width 10 ms, 20 Hz, 1.5 V, 0.63 mW or 99 Hz, 0.5 V, 0.24 mW). These parameters were titrated to produce hemodynamic responses which were similar to physiologically evoked responses. To improve the signal-to-noise for each experiment, photostimulation was presented 20–40 times in trials lasting 15–25 s each (2 s stimulation) or presented 15 times in trials lasting 70 s each (16 s stimulation).

### Electrophysiology

In order to concurrently measure hemodynamic and neural responses, in a final imaging session (occurring at least 1 week subsequent to an imaging session in which only 2D-OIS data were acquired) a 16 channel depth microelectrode (100 μm spacing, 1.5–2.7 MΩ impedance, site area 177 μm^2^; NeuroNexus Technologies) was inserted into the whisker barrel cortex through a small cranial burr hole. The electrode was positioned in the center of the whisker region (area showing the largest blood volume response to a 2 s mechanical whisker stimulation, defined by 2D-OIS in the previous imaging session) and inserted to a depth of 1500–1600 μm. The electrode was connected to both a preamplifier and data acquisition device (Medusa BioAmp/RZ5, TDT). Data were sampled at 24 kHz. For analysis, data were downsampled to 6 kHz and multiunit activity (MUA) analyzed. Data from the 12 channels covering the depth of the cortex are displayed in Figures 4, 6 and 7.

### Analysis

Analysis was performed using MATLAB (MathWorks). Using the 2D-OIS-generated spatial map of HbO_2_ response to stimulation, a region of interest (ROI) was selected for use in subsequent time-series analysis. In order to select an ROI (red ROI in Figs 3 and 5), the HbO_2_ image is processed to remove edge pixels and a Gaussian 3 × 3 filter applied. For the response period, each pixel is averaged across time to generate a mean value. The threshold for a pixel to be included in the ROI was set at 1.5 × standard deviation. Thus, the ROI represents the area with the largest hemodynamic response to the stimuli. For each experimental paradigm, the response across all pixels in the ROI was averaged in order to generate a time series for each hemodynamic profile (Hbt, HbO_2_, and Hbr). As described above, hemodynamic data were acquired with a 490 nm high pass filter, resulting in minimal light artefact from the stimulating LED. For 20 Hz stimulation, this residual artefact was removed using a high pass filter followed by a modified boxcar function, for 99 Hz stimulation, only the modified boxcar function was applied. For each experiment, mean time series for Hbt, HbO_2_, and Hbr were produced by averaging across trials. Matching experiments (e.g., 2 s photostimulation) from the same mouse on different imaging days were averaged together, so that each mouse only contributed one time series per experiment.

For the negative surround analysis, an automated “negative response” ROI was selected, with a threshold of 1.5 × standard deviation, as described above. In 3 SST-ChR2 mice in which no negative surround response was observed to either 99 or 20 Hz 16 s optogenetic stimulation, “negative response” ROIs were selected manually in an equivalent brain region.

### Statistical Analysis and Experimental Design

In order to compare hemodynamic responses, we focused on Hbt, which is our most reliable measure. Statistical comparisons were performed using GraphPad Prism. A two-way ANOVA with Tukey’s multiple comparisons test was performed in order to undertake intergroup comparisons of MUA for different electrode depths in the presence and absence of a negative surround hemodynamic response. A one-way ANOVA with Tukey’s multiple comparisons test was used to compare Hbt peaks in response with various paradigms (whisker stimulation, photostimulation of NOS-ChR2 mice, photostimulation of SST-ChR2 mice), and to compare photostimulation-evoked MUA in different mouse lines (NOS-ChR2 mice, SST-ChR2 mice, and control mice). *T*-tests (paired or unpaired, as appropriate) were used to compare between two groups (MUA in superficial vs. deep electrode channels, local field potential (LFP) deflection, and initial vs. secondary peak hemodynamic response). *P* values <0.05 were considered to be statistically significant. Data are presented as mean ± standard error in the mean (SEM). Experiments and analysis were performed unblinded to mouse type.

### Immunohistochemistry

At the end of the experiments, mice were given an overdose of pentobarbital and perfused with saline followed by 4% paraformaldehyde (PFA) in phosphate-buffered saline (PBS), via cardiac perfusion. Brains were extracted, postfixed in 4% PFA for 24 h at 4 °C, and cryoprotected in 30% sucrose in PBS at 4 °C for 48 h. The brains were sectioned using a cryostat (Thermo Fisher Scientific) to produce 30 μm coronal sections which were placed into PBS with 0.3% Triton X-100 (PBST) for 20 min to permeabilize the tissue. Free-floating sections were blocked with 10% normal donkey serum (Jackson ImmunoResearch, when staining for nNOS and EYFP) or 10% normal rabbit serum (Vector Laboratories, when staining for SST) in PBST for 1 h, and then incubated overnight at 4 °C in blocking solution with primary antibodies against GFP (anti-GFP, rabbit polyclonal, [ab290], Abcam, 1:200, recognizes EYFP, which is a genetic mutant of GFP); nNOS (anti-nNOS, goat polyclonal [ab1376], Abcam, 1:250) or SST (anti-Somatostatin, rat monoclonal [YC7] [MAB354], Millipore, 1:500). Sections were washed with PBS and incubated in the appropriate fluorescent secondary antibodies: Alexa 568 donkey anti-rabbit (Thermo Fisher Scientific, 1:500 dilution in PBS), Alexa 488 donkey anti-goat (Thermo Fisher Scientific, 1:500 dilution in PBS) for 2 h at room temperature, or in the case of the SST stain: incubated in biotinylated rabbit anti-rat (Vector Laboratories, 1:100 dilution in PBS with 1.5% normal rabbit serum) for 30 min at room temperature. The SST staining sections were then washed with PBS and incubated with Alexa 647 Streptavidin (Thermo Fisher Scientific, 1:400 dilution with PBS) for 90 min. All sections were mounted onto gelatin-coated slides following immunohistochemical staining and imaged with a fluorescence stereo microscope (M205 FA, Leica Microsystems).

## Results

### Short Duration Optogenetic Stimulation of Specific Interneurons Evokes a Localized Hemodynamic Response

**Figure 1:**
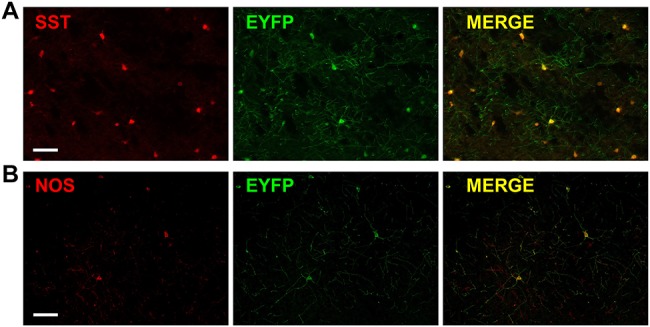
Cell-specific expression of ChR2. Immunohistochemistry showing staining for (*A*) SST (red) and ChR2 (green, EYFP is the reporter for ChR2) and merge (yellow) in cortex from SST-ChR2 mouse. (*B*) nNOS (red) and ChR2 (green, EYFP is the reporter for ChR2) and merge (yellow) in cortex from nNOS-ChR2 mouse. Scalebar represents 100 μm.

Genetically modified mice expressing channelrhodopsin-2 (ChR2) in either SST- or nNOS-expressing interneurons (referred to as SST-ChR2 or nNOS-ChR2 mice, respectively) were used to investigate how light induced activity of these inhibitory interneurons may alter cortical hemodynamics. Expression of ChR2 in the appropriate cell types was confirmed using immunohistochemistry (Fig. 1). Expression of ChR2 was evidenced by the presence of enhanced yellow fluorescent protein (EYFP), the reporter for ChR2 expression, and co-localization was seen with either SST (Fig. 1A) or nNOS (Fig. 1*B*), as appropriate. The majority of SST-positive neurons are expected to express ChR2 (Taniguchi et al. 2011). Although off-target recombination has previously been reported in the SOM-IRES-Cre mouse (Hu et al. 2013), the morphological features of observed EYFP^+^ cells (Fig. 1*A*) suggested they were SST-expressing interneurons. In the case of the nNOS-ChR2 line, in 9 representative images (from three animals), we counted 274 nNOS^+^ cells of which 248 (90.5%) were ChR2-EYFP^+^ and 26 (9.5%) were ChR2-EYFP^−^.

**Figure 2:**
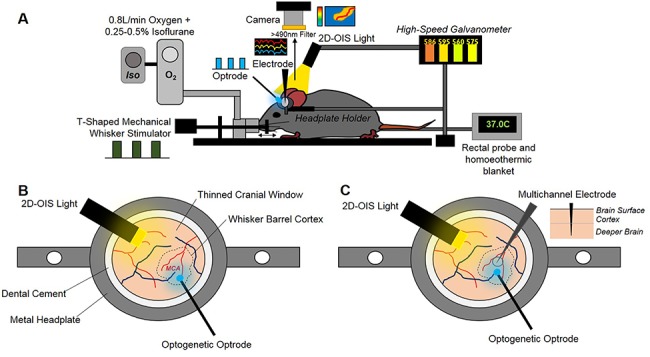
Chronic imaging preparation. (*A*) Imaging setup showing inhalational anesthetic maintenance, mechanical whisker stimulation, temperature regulation, hemodynamic imaging (2D-OIS), optogenetic stimulation, and multichannel electrode electrophysiology. (*B*) First imaging session 2-weeks post-surgery with optogenetic optrode placed over MCA/whisker barrel cortex region. (*C*) Second imaging session 3-weeks post-surgery with electrode inserted into whisker barrel cortex + optrode for optogenetic stimulation.

**Figure 3:**
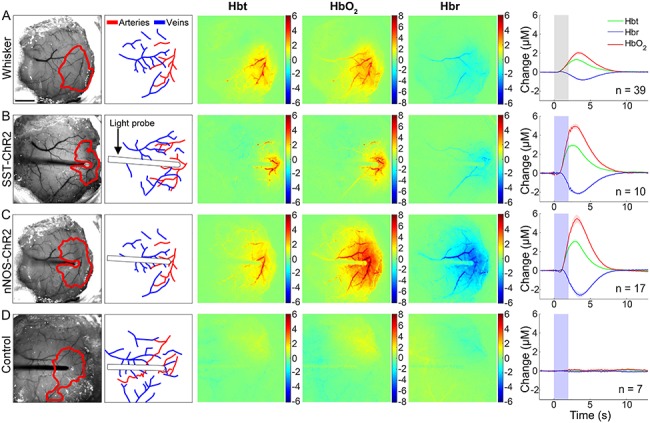
Hemodynamic responses to 2s stimulation (*A*) mechanical whisker stimulation (5 Hz), (*B*) photostimulation (99 Hz, 0.45 mW or 20 Hz, 0.78 mW) of SST-ChR2 mouse, (*C*) photostimulation of nNOS-ChR2 mice, (*D*) photostimulation of control mice. First column: representative surface vasculature overlying somatosensory cortex (at 575 nm illumination), ROI for analysis shown in red. Scalebar represents 1 mm. Second column: vessel map showing surface arteries and veins. Third, fourth, and fifth column: representative spatial activation map of trial-averaged changes in [Hbt], [HbO_2_], and [Hbr], respectively, with respect to baseline during stimulation. Color bar represents change (μM). Sixth column: mean hemodynamic time series within whisker barrel cortex ROI (mean ± SEM *n* represents number of mice). Grey box indicates whisker stimulation, blue box indicates photostimulation.

Using an anesthetized mouse (Fig. 2), we assessed whether short duration optogenetic stimulation of specific subtypes of interneuron evoked a localized hemodynamic response. 2D-OIS was used to record high-resolution 2D maps of the changes in Hbt, HbO_2_, and Hbr evoked by stimulation. Each animal initially received a mechanical whisker stimulation (2s, 5 Hz), evoking changes in Hbt, HbO_2_, and Hbr which were localized to the whisker barrel cortex (Fig. 3*A*). These hemodynamic changes allowed us to map the whisker barrel cortex and, in turn, guide the placement of the optical fiber used for photostimulation (Fig. 2). The time series of the hemodynamic response to whisker stimulation shows an increase in Hbt and HbO_2_ during the stimulation with a corresponding washout of Hbr (Fig. 3*A*).

A fiber-coupled blue (470 nm) LED, placed directly above the whisker barrel cortex, was used to apply photostimulation in order to activate ChR2-expressing neurons. The fiber optic was positioned in the center of the whisker barrel cortex (Fig. 2) and short duration photostimulation (2s: 99 Hz, 0.45 mW and 20 Hz, 0.78 mW) was applied. Similar to whisker stimulation, for both SST-ChR2 and nNOS-ChR2 mice, a short 2s stimulation produced a robust hemodynamic response focused around the tip of the fiber optic light guide. Photostimulation elicited a functional hyperemia response, showing a localized increase in Hbt and HbO_2_ with corresponding washout of Hbr (Fig. 3*B*,*C*: data from 99 and 20 Hz stimulations were combined as there was no significant difference in peak response (a one-way ANOVA showed an overall effect of stimulation type [*F*_11,113_ = 20.6, *P* < 0.0001] however, Tukey’s multiple comparisons test showed that there was no significant difference in response to stimulations which only differed in frequency of stimulation [e.g., 2s nNOS stimulation at 20 vs. 99 Hz])). Although these hemodynamic changes were similar in shape and duration to those seen in response to whisker stimulation (Fig. 3*A*), with amplitudes which were within the range of physiological responses (Gu et al. 2018), some notable differences in rise time and area under the curve were observed (see Supplementary Tables 1–3 for details). As was observed with whisker stimulation, increases in Hbt and HbO_2_ were strongest in the middle cerebral artery (MCA) branches overlaying the whisker barrel cortex with a decrease in Hbr which was clearly apparent in the draining veins (Fig. 3). When comparing peak values of the evoked Hbt response, a one-way ANOVA showed an overall effect of stimulation type (*F*_3,69_ = 51.88, *P* < 0.0001) however, Tukey’s multiple comparisons test showed that there was no significant difference in Hbt peak between SST-ChR2 and nNOS-ChR2 mice (*P* = 0.9531).

Previous studies have reported photostimulation-evoked fMRI (Christie et al. 2013) and CBF (Rungta et al. 2017) responses in optogenetically naive (non-ChR2-expressing) animals. While these studies used a laser to drive the optogenetic response, which may cause issues with heating, our light stimulation used a cold LED light source. To confirm our response was not an artefact, we applied our photostimulation paradigm to control animals (either C57bl/6J or non-ChR2-expressing littermates of nNOS-ChR2 animals) and confirmed that such stimulation failed to elicit hemodynamic responses (Fig. 3*D*).

These data demonstrate that short duration activation of specific interneuron subpopulations is sufficient to induce a localized hemodynamic response.

### Electrophysiological Response to Short Duration Stimulation is Dependent on the Specific Interneuron Population Activated

**Figure 4:**
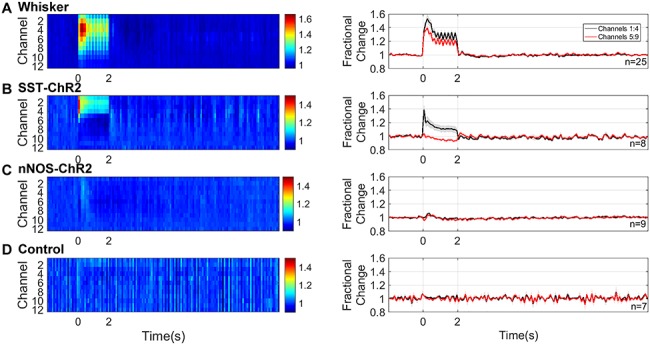
Neural responses to 2s stimulation (*A*) mechanical whisker stimulation (5 Hz), (*B*) photostimulation (99 Hz, 0.45 mW or 20 Hz, 0.78 mW) of SST-ChR2 mice, (*C*) photostimulation of nNOS-ChR2 mice, (*D*) photostimulation of control mice. First column: mean MUA response across cortical layers. Color bar represents fractional change. Second column: mean time series of response through different cortical layers. Data shown are mean ± SEM, *n* represents number of mice.

Having demonstrated that specific activation of either SST or nNOS interneurons resulted in localized hemodynamic responses, in a second experimental session, we assessed the associated evoked electrophysiological activity. Sixteen channel NeuroNexus probes were inserted into the center of the whisker barrel cortex in order to measure electrophysiological responses to both whisker stimulation and photostimulation (Fig. 2). Electrophysiological and hemodynamic measurements were made concurrently. Across all animals, 2s mechanical whisker stimulation evoked a typical electrophysiological response, with peak local MUA centered around layer 4 of the cortex. The evoked increases in MUA extended throughout the cortex (Fig. 4*A*).

Two second photostimulation of SST interneurons evoked an increase in local MUA which was limited to the superficial depth of the cortex (mean fractional change: channels 1:4 = 1.14 ± 0.05, compared with channels 5:9 = 0.96 ± 0.02, *P* = 0.0026, *n* = 8, paired *t* test, Fig. 4*B*). EEG (electroencephalogram) power band analysis (Supplementary Fig. 1) showed that photostimulation of SST interneurons resulted in an increased power both at the frequency of stimulation (20 Hz) and harmonics of it (40, 80 Hz). Such increased power in the stimulation frequency harmonic ranges has previously been reported by Ahlgrim and Manns (2019). These data confirm that photostimulation of ChR2-expressing SST interneurons results in measurable changes in neural activity in the superficial depth of the cortex.

Surprisingly, the equivalent photostimulation of ChR2-expressing nNOS interneurons elicited a minimal change in local neural activity during the light stimulation period (Fig. 4*C*). Given the robust hemodynamic response evoked by activation of nNOS interneurons (Fig. 3*C*), such a minimal change in neural activity was unexpected. This lack of robust population responses was confirmed by EEG power band analysis (Supplementary Fig. 1).

We confirmed that there were no measurable changes in neural activity evoked by photostimulation in control animals (Fig. 4*D*).

When comparing photostimulation-evoked MUA in channels 1–4 across the groups of animals, a one-way ANOVA showed an overall significant effect (*F*_2,21_ = 6.223, *P* = 0.0075). Tukey’s multiple comparisons test found that the neural activity evoked in SST-ChR2 mice was significantly different to that evoked in nNOS-ChR2 (*P* = 0.0092) and control animals (*P* = 0.0325). Taken together, our hemodynamic and electrophysiology data suggest that, in response to short duration activation, both SST and nNOS interneurons are able to evoke robust changes in blood volume and saturation. However, in the case of specific nNOS interneuron activation, the robust hemodynamic response occurred with only minimal change in local MUA.

### Long Duration Stimulation Evokes a Localized Hemodynamic Response Whose Time Course Differs Depending on the Specific Interneurons Activated

**Figure 5:**
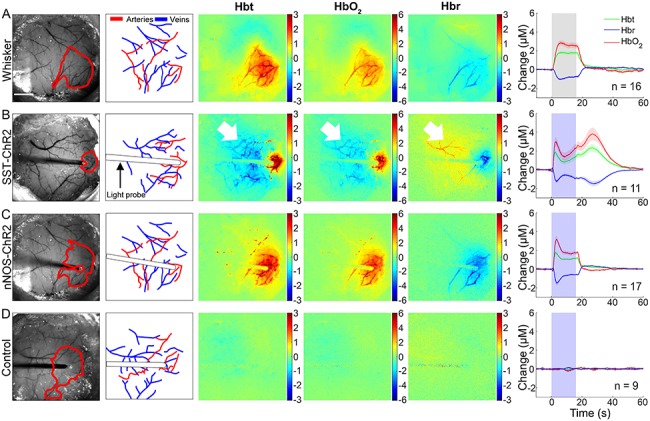
Hemodynamic responses to 16s stimulation (*A*) mechanical whisker stimulation (5 Hz), (*B*) photostimulation (99 Hz, 0.24 mW or 20 Hz, 0.63 mW) of SST-ChR2 mouse, (*C*) photostimulation of nNOS-ChR2 mice, (*D*) photostimulation of control mice. First column: representative surface vasculature overlying somatosensory cortex (at 575 nm illumination), ROI for analysis shown in red. Scalebar represents 1 mm. Second column: vessel map showing surface arteries and veins. Third, fourth, and fifth column: representative spatial activation map of trial-averaged changes in [Hbt], [HbO_2_], and [Hbr], respectively, with respect to baseline during stimulation. Color bar represents change (μM). Sixth column: mean hemodynamic time series within whisker barrel cortex ROI (mean ± SEM, *n* represents number of mice). Grey box indicates whisker stimulation, blue box indicates photostimulation. White arrowheads indicate negative surround response.

Having assessed the responses evoked by short duration interneuron stimulation, in the same group of animals we also performed long duration (16s) stimulation experiments across both modalities (whisker and photostimulation). Mechanical whisker stimulation (16s, 5 Hz) was applied and the evoked hemodynamic changes observed using 2D-OIS. Long duration whisker stimulation evoked changes in Hbt, HbO_2_, and Hbr which were localized to the whisker barrel cortex (Fig. 5*A*). The time series of the hemodynamic response to whisker stimulation shows an increase in Hbt and HbO_2_ during the stimulation period with a corresponding washout of Hbr (Fig. 5*A*). The shape of these responses are comparable with those previously reported in awake animals (Sharp et al. 2015).

LED photostimulation was performed as described above but with the stimulation period extended to 16 s (99 Hz, 0.24 mW and 20 Hz, 0.63 mW. As noted above, data were combined as no significant difference in response was observed). Specific activation of either SST (Fig. 5*B*) or nNOS (Fig. 5*C*) interneurons resulted in a localized hemodynamic response at the point of stimulation which consisted of an initial increase in Hbt and HbO_2_ with a corresponding washout of Hbr; these Hbt increases had a faster rise time and time to peak than those evoked by whisker stimulation (see Supplementary Tables 1 and 2 for details). 16s photostimulation failed to elicit hemodynamic responses in naive animals (Fig. 5*D*). When comparing peak evoked hemodynamic responses, a one-way ANOVA showed a significant effect of stimulation type (*F*_3,49_ = 12.6, *P* < 0.0001) on Hbt response. However, Tukey’s multiple comparisons test found that this was only significant in the case of photostimulation of naive animals (Hbt peak: compared to whisker stimulation, *P* < 0.0001; compared with SST activation, *P* < 0.0001; compared with nNOS activation, *P* < 0.0001).

In the case of photostimulation of SST-ChR2 mice, although the initial hemodynamic changes in the central region were similar to those in response to whisker stimulation, there were some important differences. Some SST-ChR2 animals showed a robust negative surround hemodynamic response (see white arrowheads on representative images in Fig. 5*B*), where the hemodynamic changes were the opposite of the centrally activated region. SST-ChR2 mice also showed a robust increase in blood volume and saturation after the cessation of the 16s optical stimulation, these observations will be described further. In contrast, no evidence of either a negative surround or secondary hemodynamic response was observed following specific activation of nNOS interneurons.

### Electrophysiological Response to Long Duration Stimulation is Dependent on Specific Interneuron Population Activated

**Figure 6:**
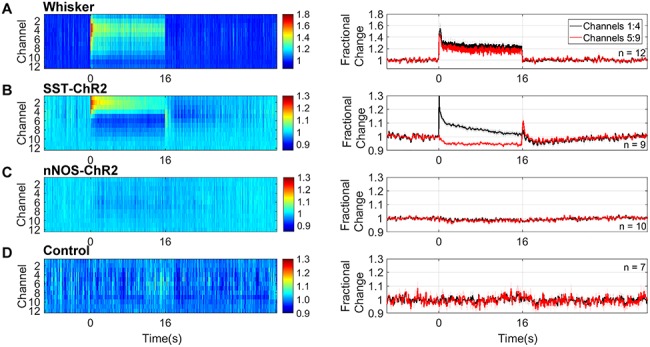
Neural responses to 16s stimulation (*A*) mechanical whisker stimulation (5 Hz), (*B*) photostimulation (99 Hz, 0.24 mW or 20 Hz, 0.63 mW) of SST-ChR2 mice, (*C*) photostimulation of nNOS-ChR2 mice, (*D*) photostimulation of control mice. First column: mean MUA response across cortical layers. Color bar represents fractional change. Second column: mean time series of response through different cortical layers. Data shown are mean ± SEM, *n* represents number of mice.

Having demonstrated that longer duration optogenetic activation of either SST- or nNOS-expressing interneurons reliably results in hemodynamic responses, in a second experimental session we assessed the associated evoked electrophysiological activity. These experiments were performed in the same group of animals as the short duration stimulation investigations. In all animals, 16s mechanical whisker stimulation evoked a typical electrophysiological response which extended throughout the cortex (Fig. 6*A*).

16s photostimulation of SST-expressing interneurons evoked an increase in local MUA in the superficial depth of the cortex and a reduction in MUA deeper in the cortex (mean fractional change in MUA: channels 1:4, 1.06 ± 0.01, *n* = 9, compared with channels 5:9, 0.95 ± 0.01, *n* = 10, *P* < 0.0001, unpaired *t* test, Fig. 6*B*). Following cessation of the photostimulation, there was a brief increase in MUA in the deeper layers, followed by a prolonged decrease below baseline in MUA which extended across cortical depth (0.97 ± 0.01 channels 1:4, 0.98 ± 0.01 channels 5:9, *P* = 0.6, unpaired *t* test). An increase in EEG power was observed at the frequency of stimulation (20 Hz) and harmonics of it (40, 80 Hz; Supplementary Fig. 2). These data confirm that specific activation of SST interneurons results in measurable changes in local neural activity in the cortex, the polarity of which is dependent on cortical depth.

An equivalent 16s photostimulation of nNOS-expressing interneurons elicited a minimal change in local neural activity during the light stimulation period (Fig. 6*C*), Although this result is surprising, given the robust hemodynamic response evoked by the photostimulation (Fig. 5*C*), it is consistent with the minimal MUA changes in response to 2s photostimulation of nNOS-expressing interneurons (Fig. 4*C*). EEG power band analysis confirmed a lack of robust population responses (Supplementary Fig. 2).

We confirmed that, as expected, no changes in neural activity were detected in response to 16s photostimulation of control animals (Fig. 6*D*).

### Long Duration Stimulation of SST-Expressing Interneurons can Evoke a Negative Surround Hemodynamic Response

**Figure 7:**
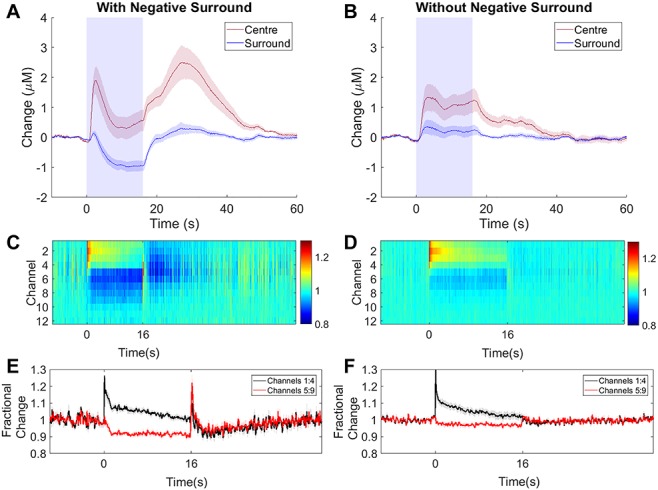
16s photostimulation elicits a negative surround in some SST-ChR2 mice. (*A*, *B*) Mean time series showing mean changes in [Hbt] with respect to baseline in SST-ChR2 mice which did (*A*, *n* = 9/16 experiments) and did not (*B*, *n* = 7/16 experiments) display a negative surround hemodynamic response. Blue box indicates photostimulation. Response in center region shown in red, surround region in blue. (*C*, *D*) Mean MUA response in central region across cortical layers for experiments which did (*C*, *n* = 9 experiments from 6 mice) and did not (*D*, *n* = 7 experiments from 6 mice) display a negative surround hemodynamic response. Color bar represents fractional change. (*E*, *F*) Mean time series of response through different cortical layers for experiments which did (*E*) and did not (*F*) display a negative surround hemodynamic response. Data shown are mean ± SEM.

When assessing the effect of long duration activation of SST interneurons, in addition to the positive hemodynamic response observed in the central activated region (surrounding the optic fiber), a negative surround hemodynamic response was observed in 9/16 experiments. The negative surround hemodynamic response, which occurs in the cortical region surrounding the central area, consisted of a decrease in Hbt and HbO_2_ and increase in Hbr (white arrowheads, Fig. 5*B*). Interestingly, the temporal dynamics of the blood volume reduction in the central region (Fig. 7*A*, red trace) were almost identical to those seen in the surround (Fig. 7*A*, blue trace). In order to further investigate the origins of the negative surround hemodynamic response, and to assess whether there was a neural marker associated with its presence, experiments in which simultaneous multi-channel electrophysiology and hemodynamic responses were recorded in the central region, were split into those in which a negative surround hemodynamic response was (Fig. 7*A*, *n* = 9 experiments, six mice) and was not (Fig. 7*B*, *n* = 7 experiments, six mice) observed in response to photostimulation. In those experiments in which a negative surround response was observed (decreased Hbt in response to photoactivation of SST neurons, Fig. 7*A*), a second increase in Hbt was also observed. This secondary hemodynamic response, which lasted for around 20 s after the photostimulation period ended, was absent in experimental sessions in which there was no negative surround hemodynamic response (Fig. 7*B*). In the central region, both the initial Hbt increase which occurred during light stimulation and the secondary Hbt increase were of similar magnitude (initial peak: 2.12 ± 0.48 μM, secondary peak: 2.81 ± 0.57 μM, *P* = 0.39, paired *t* test, Fig. 7*A*; center region).

16s photoactivation of SST interneurons evoked an increase in MUA in the superficial depth of the cortex and a decrease in MUA deeper in the cortex. The neuronal response in the superficial layers of the cortex was similar in all experiments, however, the reduction in MUA occurring in the deeper cortical layers was significantly stronger in experiments displaying a negative surround hemodynamic response (Fig. 7*C*–*F*). A two-way ANOVA showed a significant effect of both electrode channel depth (*F*_1,26_ = 59.47, *P* < 0.0001) and presence of negative surround hemodynamic response (*F*_1,26_ = 5.204, *P* = 0.031), but not for their interaction (*F*_1,26_ = 3.61, *P* = 0.0686). Tukey’s multiple comparisons test found that the change in MUA in deeper cortical layers (channels 5:9) was significantly different in the presence and absence of a negative surround hemodynamic response (*P* = 0.0253). Furthermore, in experiments which displayed a negative surround response, at the end of stimulation, there was a brief transient increase in MUA in the deeper cortical layers, followed by a long-lasting period (20s) of suppressed MUA below baseline which extended throughout the depth of the cortex (Fig. 7*C*,*E*). Neither of these phenomena were observed in experiments which were lacking a negative surround hemodynamic response (Fig. 7*D*,*F*). Further analysis of the electrophysiological data revealed the presence of a downward deflection in LFP during this “rebound” increase in MUA activity. The size of this downward deflection was significantly greater in experiments which displayed a negative surround response, compared with those which did not (0.213 ± 0.03 and 0.069 ± 0.01 V, respectively. *P* = 0.003, *t* test).

## Discussion

The present *in vivo* study investigated the contribution of two overlapping populations of GABAergic interneurons to NVC using complementary optogenetic, imaging (2D-OIS), and electrophysiological procedures. We demonstrated that photoactivation of either SST- or nNOS-expressing interneurons was sufficient to evoke a robust localized hemodynamic response. This shows that these two subsets of interneurons individually are able to drive changes in cerebral hemodynamics. In the case of specific activation of nNOS interneurons, reliable changes in hemodynamics were evoked in the presence of minimal changes in local neural activity. Meanwhile prolonged duration stimulation of SST-expressing interneurons sometimes resulted in an additional, post-stimulus, hemodynamic response, and a negative surround response which is analogous to the negative BOLD fMRI signal seen in previous studies by both our group and others (Shmuel et al. 2006; Devor et al. 2007; Boorman et al. 2010; Boorman et al. 2015). The polarity of the detected change in local neural activity evoked by SST-expressing interneurons is dependent on cortical depth, with increased MUA occurring at superficial depths and reduced MUA occurring deeper in the cortex.

Taking first the nNOS-expressing interneurons, we found that specific optical stimulation was sufficient to elicit large localized increases in blood volume and saturation. This finding confirms the general idea that GABAergic neuron activity can produce increases in blood flow (Anenberg et al. 2015; Vazquez et al. 2018) and vessel diameter (Uhlirova et al. 2016), but extends current understanding by showing that these effects can be obtained by specifically stimulating the sub-class of nNOS-expressing interneurons. We are confident that the effects we report are a consequence of the action of the LED on the channelrhodopsin in the nNOS-expressing neurons in our transgenic animals because identical optical stimulation in wild-type controls was without effect. These findings provide additional support for the proposal that nNOS interneurons play a critical role in the fundamental process of NVC (Duchemin et al. 2012). The effects in the current study are likely to be mediated through the release of NO from these neurons, which is known to be a potent vasodilator (Furchgott and Zawadzki 1980; Ignarro et al. 1987; Palmer et al. 1987).

However, perhaps the most significant finding with nNOS interneuron stimulation was that the robust hemodynamic response occurred with only a minimal change in local MUA. Compared with the effects of mild sensory stimulation where a large MUA response was associated with a moderate hemodynamic response (Figs 3*A*, 4*A*, 5*A* and 6*A*), selective stimulation of nNOS interneurons caused a larger hemodynamic response but with only a small change in MUA (Figs 3*B*, 4*B*, 5*B* and 6*B*). These findings would be consistent with a small population of optically activated nNOS interneurons (~2% of neurons in the light-activated area (Valtschanoff et al. 1993)) being responsible for the small change in MUA, yet having a dramatic effect on local hemodynamic activity. If correct, it would mean they would be in a position to play a critical role in the coupling of neural and hemodynamic activity.

While it is unlikely that nNOS interneurons play an exclusive role in NVC, these results have demonstrated for the first time that a particular subpopulation of cortical interneurons can evoke robust hemodynamic responses without being associated with the large increases in overall neural activity that would normally be expected. This observation suggests that separate populations of brain neurons, specifically nNOS interneurons, may have a disproportionate effect on cerebral hemodynamics. This suggests that any procedure or intervention that specifically targets these populations of cortical interneurons is likely to uncouple the neurovascular relationship upon which interpretations of BOLD signaling as an indirect proxy for neural activity depends.

We turn now to the case of SST-expressing interneurons. Our results show that a short 2s stimulation of SST interneurons also produced a large increase in blood volume and saturation in the activated area. With a longer duration stimulation period (16s), a longer latency negative hemodynamic response developed in adjacent unstimulated tissue (*n* = 9/16 experiments; Fig. 7), where the central positive hemodynamic response was surrounded by an inhibitory zone in adjacent tissue (Figs 5*B* and 7*A*). This inhibitory response was characterized by a marked reduction in blood volume and saturation. The temporal dynamics and magnitude of this response are similar to the negative BOLD surround region reported in previous studies by our group and others (Shmuel et al. 2006; Boorman et al. 2015). Although Uhlirova et al. (2016) have previously reported *post-stimulus*, NPY-mediated, vasoconstriction in response to optogenetic stimulation of VGAT-expressing interneurons, the present study is the first demonstration of reduced blood volume (indicative of vessel constriction) occurring *concurrently* with stimulation of a single subpopulation of cortical interneurons.

To understand better the neural basis of the center-surround pattern of hemodynamic responses elicited by stimulating SST interneurons, we performed simultaneous 2D-OIS and multi-channel electrophysiology in the central stimulated region. Given that under ostensibly constant experimental conditions, the surround hemodynamic inhibition was observed in some cases but not others, we sought to take advantage of this response variability by comparing the electrophysiological responses in the stimulated region when surround inhibition was present and when it was absent (Fig. 7). In both instances, MUA responses in the superficial cortical layers were similar. However, only in cases where the negative surround response was present, there was a strong suppression of MUA in the deep cortical layers. Then in the stimulation off-set period, after a transient increase in MUA activity, we observed a prolonged period (~20 s) of suppressed MUA below baseline across all layers.

A novel aspect of the present experiments with SST interneuron stimulation was the presence of a hemodynamic off-set response when the 16s stimulation was terminated. This effect was observed in some cases but not others. The reason for this is unclear, although slight variations in the depth of anesthesia (which can alter SST firing (Adesnik et al. 2012)) could be responsible. This variability in response may be maintained in awake animals as changes in behavior or brain state (Adesnik et al. 2012; Urban-Ciecko and Barth 2016; Yavorska and Wehr 2016) can also significantly reduce the firing rate of SST neurons. Thus, only in those cases where central photostimulation induced a surround inhibition response was the prolonged offset response in the central, previously stimulated, region observed. This later secondary peak in Hbt may be related to the long-lasting inhibition which is observed in these animals following photoactivation.

These responses during and after the 16s SST-stimulation period are difficult to interpret in terms of normal activation-induced NVC.

Firstly, there seems to be a strong association between the presence of MUA inhibition in deep cortical layers and the inhibitory hemodynamic response in surrounding tissue. How might central activation of SST interneurons reduce blood volume in surrounding regions? Karnani et al. (2016) showed that lateral inhibition between adjacent cortical regions is mediated by lateral projecting SST interneurons. We have previously shown that a decrease in deep layer MUA is correlated with the negative surround BOLD signal in rat sensory cortex (Boorman et al. 2010, 2015), our results presented here are qualitatively similar, suggesting SST interneurons projecting into neighboring regions may be responsible. However, as SST neurons can inhibit pyramidal neurons and other inhibitory neurons (as reviewed by Yavorska and Wehr 2016) and these experiments were performed without the use of pharmacological blockers, we cannot rule out the possibility that the observed negative surround hemodynamic response is a result of suppression of excitatory transmission (Urban-Ciecko and Barth 2016).

Secondly, there was a similarly strong association between reductions in deep-layer MUA and the occurrence of a large offset hemodynamic response in the previously stimulated region. It is particularly important to note that the positive post-stimulus hemodynamic response occurred without any corresponding long-lasting increase in MUA. Indeed, the central area MUA was suppressed during this positive hemodynamic response. In light of our findings with the nNOS interneurons (see above), one possibility could be that following SST interneuron stimulation offset, the small population of nNOS cells in the central stimulated region become active, potentially due to removal of inhibition from the activated SST interneurons, thereby causing the large hemodynamic response during a period of overall MUA suppression (Fig. 7*C*,*E*). Alternatively, Mariotti et al. (2018) reported that activation of cortical SST interneurons caused delayed long-lasting [Ca^2+^]_i_ elevations in astrocytes. Increased astrocyte [Ca^2+^]_i_ is known to produce significant vasodilation (Zonta et al. 2003; Gordon et al. 2008; Lind et al. 2013), therefore SST interneuron-evoked astrocyte [Ca^2+^]_i_ increases could cause the observed positive post-stimulus hemodynamic response. However, whether the time course of SST activation of astrocytes accords with the other neural and hemodynamic changes observed here remains to be determined.

On the other hand, the positive post-stimulus hemodynamic response may be associated with the brief post-stimulus transient increase in MUA activity. This activity occurs at a deeper depth in the cortex than the SST activation-evoked superficial increase in MUA, suggesting that a different population of neurons may be responsible for this MUA “rebound”. The observation of a concurrent downward deflection in the LFP suggests that pyramidal cells may be involved in the observed “rebound” activity (Bean 2007; Uhlirova et al. 2016).

Given that up to 40% of SST neurons co-express nNOS (Yavorska and Wehr 2016), it is likely that a subset of neurons is activated in response to both nNOS-ChR2 and SST-ChR2 activation. Based on previous reports (Perrenoud et al. 2012), the overlapping population of SST^+^/nNOS^+^ neurons are likely to be located in layer VI of the cortex and to be predominantly type I nNOS interneurons (which are strongly labelled for nNOS and have high expression levels of SST, conversely only around 18% of type II nNOS neurons co-express SST). This suggests that NO release could, at least in part, be responsible for the SST-evoked hemodynamic responses. Interestingly, although nNOS interneurons comprise a smaller number of neurons than SST interneurons, we observed hemodynamic responses of similar amplitude during activation of either SST or nNOS-expressing neurons. This could be explained by the fact that NO is a potent vasodilator (Furchgott and Zawadzki 1980; Ignarro et al. 1987; Palmer et al. 1987). On the other hand, as the latent increase in Hbt is only observed in the case of 16s SST activation, it is likely that this response is somehow SST-dependent. Future pharmacological studies could determine the contribution of each of these neuronal sub-populations to neurovascular regulation and confirm whether NO is indeed contributing to the observed hemodynamic responses.

While the use of anesthesia in this study may be considered a limitation (Gao et al. 2017), we have previously demonstrated that in our chronic preparation (as used in the present study), mechanical somatosensory-evoked hemodynamic responses are comparable with those observed in the awake animal (Sharp et al. 2015). Furthermore, hemodynamic responses evoked by the activation of VGAT-expressing neurons exhibit similar amplitudes and time courses in anesthetized and awake animals (Uhlirova et al. 2016). Therefore, we expect that activating SST or nNOS interneurons (subsets of the VGAT population) in an awake animal would evoke similar hemodynamic responses to those reported here.

Overall, the results of this study extend our knowledge of how specific subpopulations of cortical GABAergic interneurons mediate key aspects of neurovascular control. This has implications for our understanding of several diseases in which NVC and inhibitory interneurons are dysfunctional or lost; including epilepsy (Dudek and Shao 2003; Kumar and Buckmaster 2006; Harris et al. 2013) and Alzheimer’s disease (Zlokovic 2010; Verret et al. 2012). Indeed, the demonstration that the targeting of a single cell population reliably evokes robust hemodynamic changes in the absence of associated large increases in neural activity suggests a potential novel treatment strategy for diseases in which chronic hypoperfusion plays a role, such as Alzheimer’s disease. Meanwhile, the novel demonstration of deep layer inhibition during specific activation of SST interneurons could potentially be used to shut down aberrant neuronal activity, thus offering therapeutic strategies for diseases such as epilepsy. Future work should focus on the relationship between interneuron deficits, dysfunctional NVC, and disease progression.

## Funding

Wellcome Trust and Royal Society Sir Henry Dale Fellowship (grant number 105586/Z/14/Z to C.H.); Medical Research Council UK (grant number MR/M013553/1 to J.B and L.B).

## Notes

We would like to thank Michael Port for building and maintaining the whisker stimulation device and 2D-OIS apparatus.

## Supplementary Material

Tables1_2_3_bhz251

Sup_fig_1_bhz251

Sup_fig_2_bhz251
