# Supplementary material for: Key Aspects of Neurovascular Control Mediated by Specific Populations of Inhibitory Cortical Interneurons
Source: Cereb Cortex. 2019 Nov 20;30(4):2452–64. doi: 10.1093/cercor/bhz251 (PMC7174996; doi:10.1093/cercor/bhz251)
Supplement: Tables1_2_3_bhz251 [file tables1_2_3_bhz251.docx]

**Supplementary** **Table 1: Rise time of Hbt**

| **Stimulation paradigm** | **Rise time (s) [mean ± s.e.m]** | **n (mice)** |
| --- | --- | --- |
| 2s whisker | 1.68 ± 0.04 | 39 |
| 2s nNOS | 1.12 ± 0.07 | 17 |
| 2s SST | 0.89 ± 0.09 | 10 |
| 16s whisker | 3.06 ± 0.21 | 16 |
| 16s nNOS | 1.13 ± 0.11 | 17 |
| 16s SST | 0.77 ± 0.11 | 11 |

Photostimulation-evoked a significantly shorter Hbt rise time (taken as rise time between 10-85% of first peak response) than that evoked by whisker stimulation. When comparing rise time of evoked Hbt responses, for 2s stimulation a one-way ANOVA showed an overall effect of stimulation type (F_2,63_ =62.73, p<0.0001). Tukey’s multiple comparisons test revealed that there was a significant difference between whisker stimulation-evoked responses and both NOS-activation (p<0.0001) and SST-activation (p<0.0001) responses and between NOS and SST-evoked responses (p = 0.044).

For 16s stimulation, a one-way ANOVA showed a significant effect of stimulation type (F_2,41_ =59.61, p<0.0001) on 16s stimulation-evoked Hbt response. Tukey’s multiple comparisons test found a significant difference between whisker stimulation and NOS (p<0.0001) or SST-activation (p<0.0001).

**Supplementary Table 2: Time to peak, Hbt**

| **Stimulation paradigm** | **Time to peak (s, mean ± s.e.m)** | **n (mice)** |
| --- | --- | --- |
| 2s whisker | 2.61 ± 0.05 | 39 |
| 2s nNOS | 2.00 ± 0.08 | 17 |
| 2s SST | 1.71 ± 0.11 | 10 |
| 16s whisker | 4.26 ± 0.17 | 16 |
| 16s nNOS | 2.12 ± 0.16 | 17 |
| 16s SST | 1.71 ± 0.24 | 11 |

Photostimulation-evoked a significantly shorter time to peak Hbt than that evoked by whisker stimulation. When comparing time to peak of the Hbt time series, for 2s stimulation a one-way ANOVA showed an overall effect of stimulation type (F_2,63_ =47.56, p<0.0001). Tukey’s multiple comparisons test revealed that there was a significant difference between whisker stimulation-evoked responses and both NOS-activation (p<0.0001) and SST-activation (p<0.0001) responses.

For 16s stimulation, a one-way ANOVA showed a significant effect of stimulation type (F_2,41_ =57.2, p<0.0001) on 16s stimulation-evoked Hbt response. Tukey’s multiple comparisons test revealed that there was a significant difference between whisker stimulation-evoked responses and both NOS-activation (p<0.0001) and SST-activation (p<0.0001) responses.

**Supplementary Table 3: Area under curve, Hbt**

| **Stimulation paradigm** | **Area under curve (mean ± s.e.m)** | **n (mice)** |
| --- | --- | --- |
| 2s whisker | 5.65 ± 0.42 | 39 |
| 2s nNOS | 10.7 ± 0.58 | 17 |
| 2s SST | 11.38 ± 0.73 | 10 |
| 16s whisker | 41.73 ± 4.56 | 16 |
| 16s nNOS | 28.27 ± 2.41 | 17 |
| 16s SST | 64.79 ± 8.05 | 11 |

When comparing area under curve of the Hbt time series, for 2s stimulation a one-way ANOVA showed an overall effect of stimulation type (F_2,63_ =35.86, p<0.0001). Tukey’s multiple comparisons test revealed that there was a significant difference between whisker stimulation-evoked responses and both NOS-activation (p<0.0001) and SST-activation (p<0.0001) responses.

For 16s stimulation, a one-way ANOVA showed a significant effect of stimulation type (F_2,41_ =13.35, p<0.0001) on 16s stimulation-evoked Hbt response. However, in this case, Tukey’s multiple comparisons test found a significant difference between whisker stimulation and SST activation (p = 0.007) and between NOS and SST-activation (p<0.0001).
