# Supplementary figures and images for: Key Aspects of Neurovascular Control Mediated by Specific Populations of Inhibitory Cortical Interneurons
Source: Cereb Cortex. 2019 Nov 20;30(4):2452–64. doi: 10.1093/cercor/bhz251 (PMC7174996; doi:10.1093/cercor/bhz251)

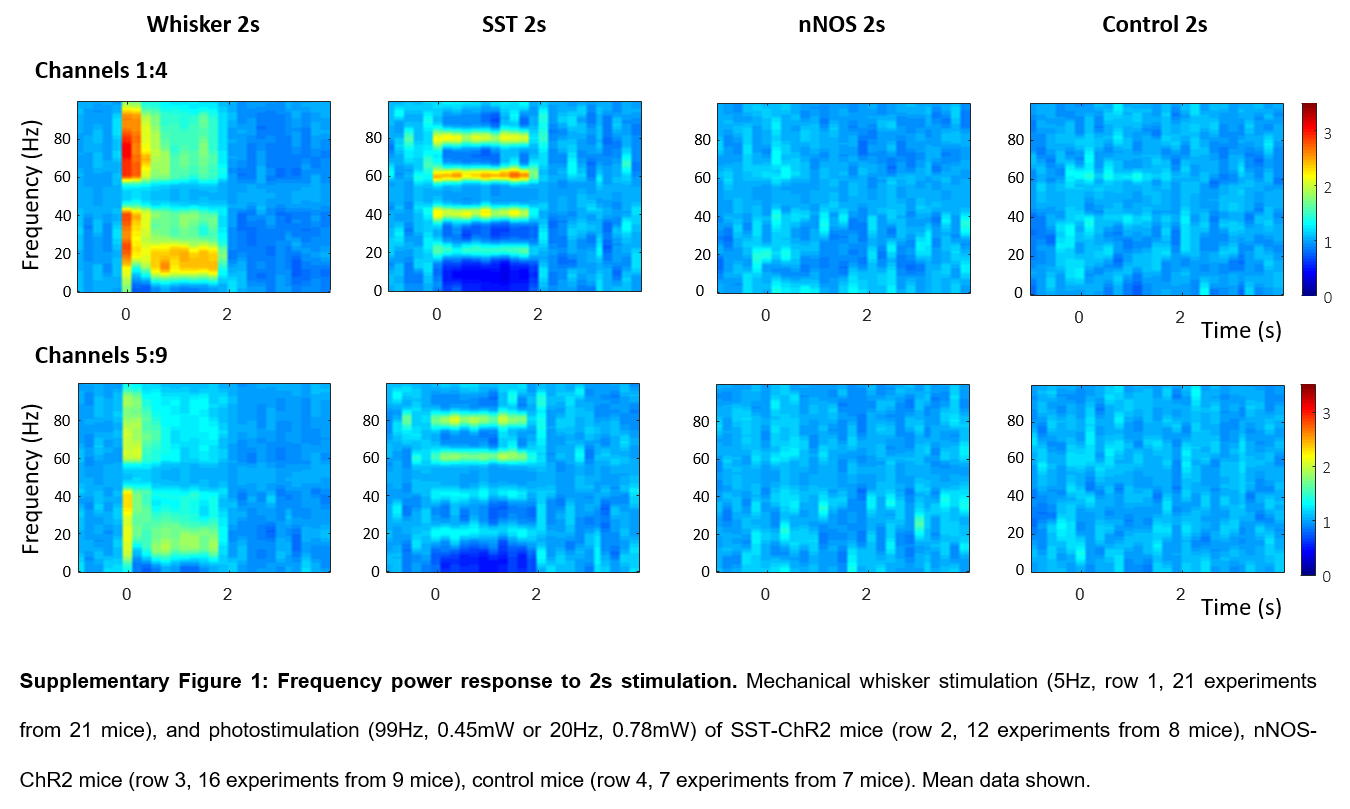

Supplement: Sup_fig_1_bhz251 [file sup_fig_1_bhz251.png]

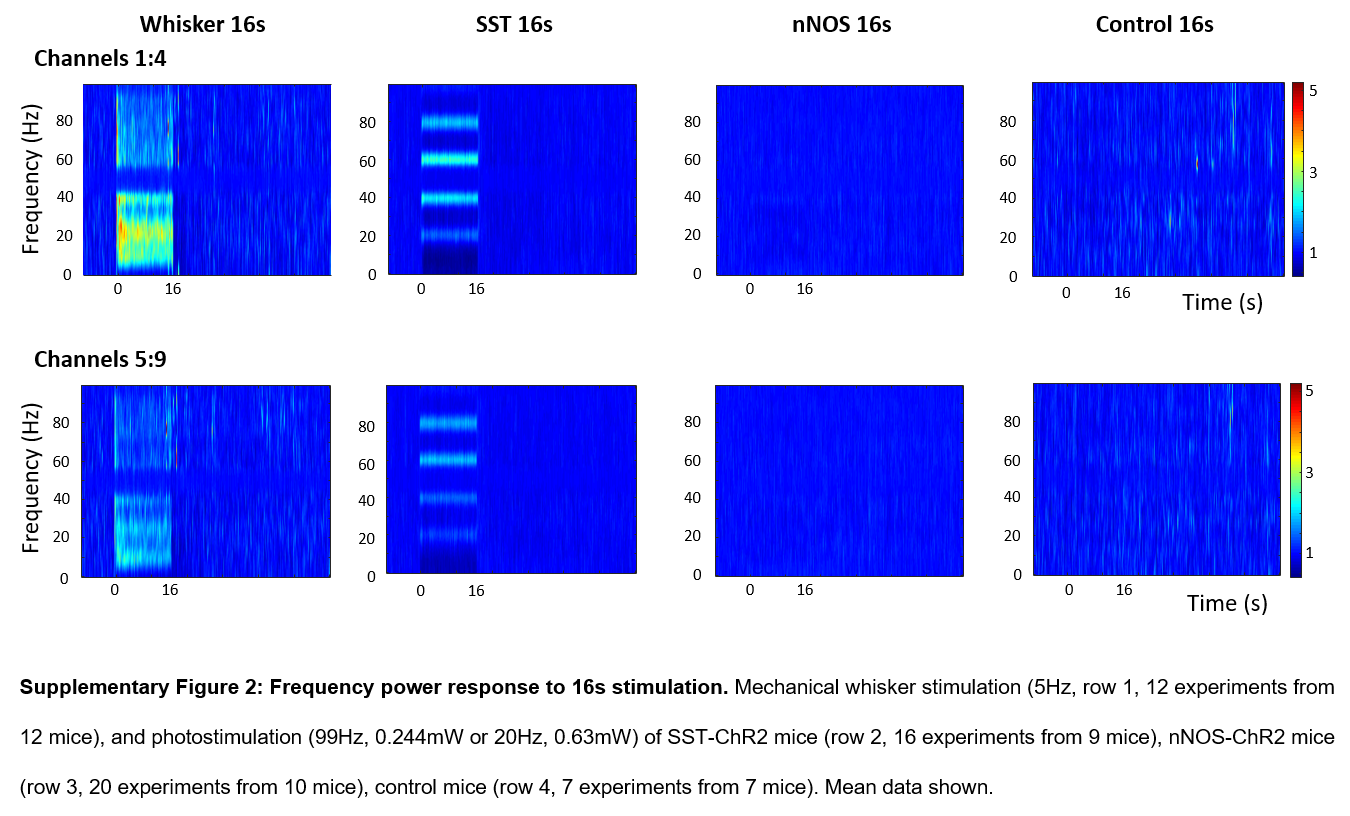

Supplement: Sup_fig_2_bhz251 [file sup_fig_2_bhz251.png]
